# Supplementary material for: Genomic Analysis of Two MDR Isolates of Salmonella enterica Serovar Infantis from a Spanish Hospital Bearing the blaCTX-M-65 Gene with or without fosA3 in pESI-like Plasmids
Source: Antibiotics (Basel). 2022 Jun 9;11(6):786. doi: 10.3390/antibiotics11060786 (PMC9219668; doi:10.3390/antibiotics11060786)
Supplement: Supplementary file 1 [file antibiotics-11-00786-s001.zip › antibiotics-1745783-supplementary.pdf]

Supplementary Material

Genomic analysis of two MDR isolates of *Salmonella enterica* serovar Infantis from a Spanish hospital bearing the *bla*$_{CTX-M-65}$ gene with and without *fosA3* in pESI-like plasmids

Table S1. Accession numbers of the genomes of two cefotaxime resistance isolates of *Salmonella enterica* serovar Infantis, and parameters related to the quality of the assemblies

Isolate[a]	kmer	Contigs	N50	Longest contig (bp)	Total bp in contigs	Contigs > 1 kb	Library	Coverage	GenBank accession number
HUMV 13-6278	93	88	245,837	1,195,887	5,000,585	44	516 +/- 130	26x	JAICDN000000000
HUMV 15-5476	91	86	245,896	1,195,313	4,998,779	44	525 +/- 130	22x	JAICDM000000000

[a], HUMV, "Hospital Universitario Marqués de Valdecillas", Cantabria, Santander, Spain. Isolates are designated with the initials of the hospital, followed by the last two numbers of the year of recovery-serial number.

Table S2. Origin and accession numbers of the genomes of *Salmonella enterica* serovar Infantis isolates used for phylogenetic analysis in the present study

Isolate	Sample [a]	Country	Year of isolation	*bla*CTX-M / *fosA3* [b]	GenBank/ENA accession no. [c]	Reference
119944	Human feces	Israel	2008	- / -	GCA_000506925.1	[1]
MRS-16/01939	Broiler flock	Austria	2020	- / -	SAMN19328299	[2]
MRS-17/00712	Broiler flock	Austria	2020	- / -	SAMN19328300	[2]
17-01817	Human feces	Germany	2017	*bla*CTX-M-1 / -	ERX4702755 [c]	[3]
17-04725	Human feces	Germany	2017	*bla*CTX-M-1 / -	ERX4702767 [c]	[3]
18-01586	na	Germany	2018	*bla*CTX-M-1 / -	ERX470278 [c]	[3]
18-01982	Human feces	Germany	2018	*bla*CTX-M-1 / -	ERX4702792 [c]	[3]
18-02671	Human feces	Germany	2018	*bla*CTX-M-1 / -	ERX4702799 [c]	[3]
18-03381	Human feces	Germany	2018	*bla*CTX-M-1 / -	ERX4702804 [c]	[3]
18-04022	Human feces	Germany	2018	*bla*CTX-M-1 / -	ERX4702810 [c]	[3]
18-06875	na	Germany	2018	*bla*CTX-M-1 / -	ERX4702828*	[3]
18-06974	Human feces	Germany	2018	*bla*CTX-M-1 / -	ERX4702829 [c]	[3]
19-00103	Human feces	Germany	2019	*bla*CTX-M-1 / -	ERX4702832 [c]	[3]
19-01785	Human feces	Germany	2019	*bla*CTX-M-1 / -	ERX4702844 [c]	[3]
19-03124	Human feces	Germany	2019	*bla*CTX-M-1 / -	ERX4702861 [c]	[3]
19-04093	Human feces	Germany	2019	*bla*CTX-M-1 / -	ERX4702868 [c]	[3]
19-04501	na	Germany	2019	*bla*CTX-M-1 / -	ERX4702875 [c]	[3]
19-04642	Human feces	Germany	2019	*bla*CTX-M-1 / -	ERX4702878 [c]	[3]
16092401-41	Broiler chicken	Italy	2016	*bla*CTX-M-1 / -	ERS2521096 [c]	[4]
16092401-42	Broiler chicken	Italy	2016	*bla*CTX-M-1 / -	ERS2521097 [c]	[4]
16072017	Broiler chicken	Italy	2016	- / -	ERS2030111 [c]	[4]
17095712-68	Broiler meat	Italy	2017	- / -	ERS2521098 [c]	[4]
14026835	Human (na)	Italy	2014	*bla*CTX-M-65 / +	ERR1014119 [c]	[5]
1091300903	Human feces	The Netherlands	2013	*bla*CTX-M-65 / +	ERS2030077 [c]	[6]
1091400879	Human (na)	The Netherlands	2014	*bla*CTX-M-65 / +	ERS2030079 [c]	[6]
1091401725	Human feces	The Netherlands	2014	*bla*CTX-M-65 / +	ERS2030086 [c]	[6]

1091600414	Human (na)	The Netherlands	2016	$bla_{\text{CTX-M-65}}$ / +	ERS2030074 [c]	[6]
1091603069	Human (na)	The Netherlands	2017	$bla_{\text{CTX-M-65}}$ / +	ERS2030068 [c]	[6]
91264	Human (na)	England and Wales	2015	- / -	CP070301.1	[7]
N55391	Chicken breast	USA	2014	$bla_{\text{CTX-M-65}}$ / -	GCA_001931595.1	[8]
18TX11CB22-S1	Chicken breast	USA	2018	$bla_{\text{CTX-M-65}}$ / -	CP082538 - CP082539	[9]
CVM N18S2085	Chicken wings	USA	2018	- / -	CP052776.1	[10]
CVM N18S2198	Chicken breast	USA	2018	$bla_{\text{CTX-M-65}}$ / -	CP082521 - CP082522	[9]
CVM N19S0949	Chicken wings	USA	2019	- / -	CP052780.1	[10]
CFSAN003307	Creek water	USA	2011	- / -	GCA_002863785.1	[1]
FSIS1502169	Chicken ceca	USA	2015	$bla_{\text{CTX-M-65}}$ / +	CP016406 - CP016407	[8]
FSIS1502916	Comminuted chicken	USA	2015	$bla_{\text{CTX-M-65}}$ / +	GCA_001931575.1	[8]
CVM44454	Human (na)	USA	2014	$bla_{\text{CTX-M-65}}$ / -	GCA_001931615.1	[8]
FARPER-219	Chicken liver and spleen	Peru	2017	$bla_{\text{CTX-M-65}}$ / +	GCA_006402875.1	[11]
CFSAN103796	Cloacae swab	Trinidad and Tobago	2019	$bla_{\text{CTX-M-65}}$ / -	GCA_012957765.2	[12]
TR01	Poultry flock	Turkey	2017	- / -	CP040600.1	[13]
HUMV 13-6278	Human feces	Spain	2013	$bla_{\text{CTX-M-65}}$ / -	JAICDN000000000	This study
HUMV 15-5476	Human feces	Spain	2015	$bla_{\text{CTX-M-65}}$ / +	JAICDM000000000	This study

[a], na, information not available.
[b], -, absent.
[c], The raw reads of the genome sequence were retrieved from the ENA (European Nucleotide Archive; http://www.ebi.ac.uk/ena) and assembled by using VelvetOptimiser.pl script implemented in the "on line" version of PLACNETw (https://omictools.com/placnet-tool. When two accession numbers are indicated they correspond to the chromosome and the pESI-like plasmids of the isolate.

References

1. Cohen, E, Rahav, G, and Gal-Mor, O. Genome Sequence of an Emerging *Salmonella enterica* Serovar Infantis and Genomic Comparison with Other *S.* Infantis Strains. *Genome Biol. Evol.* **2020,** 12(3): 151-159. doi: 10.1093/gbe/evaa048
2. Drauch, V.; Kornschober, C.; Palmieri, N.; Hess, M.; Hess, C. Infection dynamics of *Salmonella* Infantis strains displaying different genetic backgrounds - with or without pESI-like plasmid - vary considerably. *Emerg. Microbes. Infect.* **2021,** *10*, 1471–1480, doi:10.1080/22221751.2021.1951124
3. Pietsch, M.; Simon, S.; Meinen, A.; Trost, E.; Banerji, S.; Pfeifer, Y.; Flieger, A. Third generation cephalosporin resistance in clinical non-typhoidal *Salmonella enterica* in Germany and emergence of $bla_{\text{CTX-M}}$-harbouring pESI plasmids. *Microb. Genom.* **2021,** 7, doi:10.1099/mgen.0.000698
4. Carfora, V.; Alba, P.; Leekitcharoenphon, P.; Ballaro, D.; Cordaro, G.; Di Matteo, P.; Donati, V.; Ianzano, A.; Iurescia, M.; Stravino, F.; et al. Colistin Resistance Mediated by *mcr-1* in ESBL-Producing, Multidrug Resistant *Salmonella* Infantis in Broiler Chicken Industry, Italy (2016-2017). *Front. Microbiol.* **2018,** *9*, 1880, doi: 10.3389/fmicb.2018.01880

5. Franco, A, Leekitcharoenphon, P, Feltrin, F, Alba, P, Cordaro, G, Iurescia, M, Tolli, R, D'Incau, M, Staffolani, M, Di Giannatale, E, Hendriksen, RS, and Battisti, A. Emergence of a Clonal Lineage of Multidrug-Resistant ESBL-Producing *Salmonella* Infantis Transmitted from Broilers and Broiler Meat to Humans in Italy between 2011 and 2014. *PLoS One*. **2015**, 10(12): e0144802. doi: 10.1371/journal.pone.0144802
6. Alba, P, Leekitcharoenphon, P, Carfora, V, Amoruso, R, Cordaro, G, Di Matteo, P, Ianzano, A, Iurescia, M, Diaconu, EL, Study Group, EN, Pedersen, SK, Guerra, B, Hendriksen, RS, Franco, A, and Battisti, A. Molecular epidemiology of *Salmonella* Infantis in Europe: insights into the success of the bacterial host and its parasitic pESI-like megaplasmid. *Microb. Genom.* **2020**, 6(5). doi: 10.1099/mgen.0.000365
7. Lee, WWY, Mattock, J, Greig, DR, Langridge, GC, Baker, D, Bloomfield, S, Mather, AE, Wain, JR, Edwards, AM, Hartman, H, Dallman, TJ, Chattaway, MA, and Nair, S. Characterization of a pESI-like plasmid and analysis of multidrug-resistant *Salmonella enterica* Infantis isolates in England and Wales. *Microb. Genom.* **2021**, 7(10). doi: 10.1099/mgen.0.000658
8. Tate, H, Folster, JP, Hsu, CH, Chen, J, Hoffmann, M, Li, C, Morales, C, Tyson, GH, Mukherjee, S, Brown, AC, Green, A, Wilson, W, Dessai, U, Abbott, J, Joseph, L, Haro, J, Ayers, S, McDermott, PF, and Zhao, S. Comparative Analysis of Extended-Spectrum-β-Lactamase CTX-M-65-Producing *Salmonella enterica* Serovar Infantis Isolates from Humans, Food Animals, and Retail Chickens in the United States. *Antimicrob. Agents Chemother.* **2017**, 61(7). doi: 10.1128/AAC.00488-17
9. Li, C, Tyson, GH, Hsu, CH, Harrison, L, Strain, E, Tran, TT, Tillman, GE, Dessai, U, McDermott, PF, and Zhao, S. Long-Read Sequencing Reveals Evolution and Acquisition of Antimicrobial Resistance and Virulence Genes in *Salmonella enterica*. *Front. Microbiol.* **2021**, 12: 777817. doi: 10.3389/fmicb.2021.777817
10. Tyson, GH, Li, C, Harrison, LB, Martin, G, Hsu, CH, Tate, H, Tran, TT, Strain, E, and Zhao, S. A Multidrug-Resistant *Salmonella* Infantis Clone is Spreading and Recombining in the United States. *Microb. Drug Resist.* **2021**, 27(6): 792-799. doi: 10.1089/mdr.2020.0389
11. Vallejos-Sanchez, K, Tataje-Lavanda, L, Villanueva-Perez, D, Bendezu, J, Montalvan, A, Zimic-Peralta, M, Fernandez-Sanchez, M, and Fernandez-Diaz, M. Whole-Genome Sequencing of a *Salmonella enterica subsp. enterica* Serovar Infantis Strain Isolated from Broiler Chicken in Peru. *Microbiol. Resour. Announc.* **2019**, 8(43). doi: 10.1128/MRA.00826-19
12. Maguire, M, Khan, AS, Adesiyun, AA, Georges, K, and Gonzalez-Escalona, N. Genomic comparison of eight closed genomes of multidrug resistant *Salmonella enterica* strains isolated from broiler farms and processing plants in Trinidad and Tobago. *Front. Microbiol.* **2022**, *13*, 863104. https://doi.org/10.1101/2021.05.20.445003.
13. Müstak, I.B.; Yardimci, H. Construction and in vitro characterisation of *aroA* defective (*aroAΔ*) mutant *Salmonella* Infantis. *Arch. Microbiol.* **2019**, *201*, 1277-1284, doi: 10.1007/s00203-019-01694-0.

Table S3. Pairwise distance matrix calculated from SNP in the genomes of two *Salmonella enterica* serovar Infantis human isolates from a Spanish hospital and 41 isolates from other sources and countries.

	1091300903	1091400879	1091401725	1091600414	1091603069	119944	14026835	16072017	16092401-41	17-0181	17-04725	17095712-68	18-01586	18-01982	18-02671	18-03381	18-04022	18-06875	18-06974	18TX11CB22-S1	19-00103	19-01785	19-03124	19-04093	19-04501	19-04642	6092401-42	91264	CFSAN003307	CFSAN103796	CVM44454	CVM N1852085	CVM N1852198	CVM N1950949	FARPER-219	FSIS1502169	FSIS1502916	HUMV 13-6278	HUMV 15-5476	MRS-16/01939	MRS-17/00712	N55391	TR01
**1091300903**	0	15	22	32	32	97	25	123	131	113	161	126	163	148	134	155	122	158	156	36	160	178	170	126	153	196	132	123	164	48	18	144	39	38	27	24	26	14	20	147	257	23	251
**1091400879**	15	0	17	27	27	94	22	120	128	110	158	123	160	145	131	152	119	155	153	31	157	175	167	123	150	193	129	120	161	43	13	141	34	33	22	19	21	11	17	144	254	18	248
**1091401725**	22	17	0	34	34	101	29	127	135	117	165	130	167	152	138	159	126	162	160	38	164	182	174	130	157	200	136	127	168	50	20	148	41	40	29	26	28	18	24	151	261	25	255
**1091600414**	32	27	34	0	0	111	39	137	145	127	175	140	177	162	148	169	136	172	170	48	174	192	184	140	167	210	146	137	178	60	30	158	51	50	39	36	38	28	34	161	271	35	265
**1091603069**	32	27	34	0	0	111	39	137	145	127	175	140	177	162	148	169	136	172	170	48	174	192	184	140	167	210	146	137	178	60	30	158	51	50	39	36	38	28	34	161	271	35	265
**119944**	97	94	101	111	111	0	100	112	118	100	148	113	150	135	123	142	109	145	143	115	147	165	157	113	140	183	119	110	151	127	97	131	118	117	106	103	105	93	99	134	246	102	238
**14026835**	25	22	29	39	39	100	0	126	134	116	164	129	166	151	137	158	125	161	159	43	163	181	173	129	156	199	135	126	167	55	25	147	46	45	34	31	33	21	27	150	260	30	254
**16072017**	123	120	127	137	137	112	126	0	72	54	174	17	176	161	149	168	63	171	169	141	173	191	183	139	166	209	73	136	177	153	123	157	144	143	132	129	131	119	125	160	272	128	264
**16092401-41**	131	128	135	145	145	118	134	72	0	28	180	73	182	167	155	174	35	177	175	149	177	197	187	145	172	213	43	142	183	161	131	163	152	151	140	137	139	127	131	166	276	136	270
**17-0181**	113	110	117	127	127	100	116	54	28	0	162	55	164	149	137	156	19	159	157	131	161	179	171	127	154	197	29	124	165	143	113	145	134	133	122	119	121	109	115	148	260	118	252
**17-04725**	161	158	165	175	175	148	164	174	180	162	0	175	128	91	101	98	171	123	121	179	125	143	137	125	118	161	181	122	119	191	161	83	182	181	170	167	169	157	163	194	242	166	238
**17095712-68**	126	123	130	140	140	113	129	17	73	55	175	0	177	162	150	169	64	172	170	144	174	192	184	140	167	210	74	137	178	156	126	158	147	146	135	132	134	122	128	161	273	131	265
**18-01586**	163	160	167	177	177	150	166	176	182	164	128	177	0	115	103	122	173	39	37	181	41	57	55	127	34	77	183	124	131	193	163	111	184	183	172	169	171	159	165	196	244	168	240
**18-01982**	148	145	152	162	162	135	151	161	167	149	91	162	115	0	88	13	158	110	108	166	112	130	124	112	105	148	168	109	106	178	148	74	169	168	157	154	156	144	150	181	229	153	225
**18-02671**	134	131	138	148	148	123	137	149	155	137	101	150	103	88	0	95	146	98	96	152	100	118	112	100	93	136	156	97	104	164	134	84	155	154	143	140	142	130	136	169	217	139	213
**18-03381**	155	152	159	169	169	142	158	168	174	156	98	169	122	13	95	0	165	117	115	173	119	137	131	119	112	155	175	116	113	185	155	81	176	175	164	161	163	151	157	188	236	160	232
**18-04022**	122	119	126	136	136	109	125	63	35	19	171	64	173	158	146	165	0	168	166	140	170	188	180	136	163	206	36	133	174	152	122	154	143	142	131	128	130	118	124	157	269	127	261
**18-06875**	158	155	162	172	172	145	161	171	177	159	123	172	39	110	98	117	168	0	10	176	14	52	28	122	7	50	178	119	126	188	158	106	179	178	167	164	166	154	160	191	239	163	235
**18-06974**	156	153	160	170	170	143	159	169	175	157	121	170	37	108	96	115	166	10	0	174	12	50	26	120	5	48	176	117	124	186	156	104	177	176	165	162	164	152	158	189	237	161	233
**18TX11CB22-S1**	36	31	38	48	48	115	43	141	149	131	179	144	181	166	152	173	140	176	174	0	178	196	188	144	171	214	150	141	182	24	34	162	31	10	43	16	18	32	38	165	275	15	269
**19-00103**	160	157	164	174	174	147	163	173	177	161	125	174	41	112	100	119	170	14	12	178	0	54	26	124	9	44	180	121	128	190	160	108	181	180	169	166	168	156	160	191	237	165	237
**19-01785**	178	175	182	192	192	165	181	191	197	179	143	192	57	130	118	137	188	52	50	196	54	0	68	142	47	90	198	139	146	208	178	126	199	198	187	184	186	174	180	211	259	183	255
**19-03124**	170	167	174	184	184	157	173	183	187	171	137	184	55	124	112	131	180	28	26	188	26	68	0	136	23	50	190	133	140	200	170	120	191	190	179	176	178	166	170	208	251	175	249
**19-04093**	126	123	130	140	140	113	129	139	145	127	125	140	127	112	100	119	136	122	120	144	124	142	136	0	117	160	146	87	128	156	126	108	147	146	135	132	134	122	128	159	223	131	219
**19-04501**	153	150	157	167	167	140	156	166	172	154	118	167	34	105	93	112	163	7	5	171	9	47	23	117	0	45	173	114	121	183	153	101	174	173	162	159	161	149	155	186	234	158	230
**19-04642**	196	193	200	210	210	183	199	209	213	197	161	210	77	148	136	155	206	50	48	214	44	90	50	160	45	0	216	157	164	226	196	144	217	216	205	202	204	192	194	227	273	201	273
**6092401-42**	132	129	136	146	146	119	135	73	43	29	181	74	183	168	156	175	36	178	176	150	180	198	190	146	173	216	0	143	184	162	132	164	153	152	141	138	140	128	134	167	279	137	271
**91264**	123	120	127	137	137	110	126	136	142	124	122	137	124	109	97	116	133	119	117	141	121	139	133	87	114	157	143	0	125	153	123	105	144	143	132	129	131	119	125	156	220	128	216
**CFSAN003307**	164	161	168	178	178	151	167	177	183	165	119	178	131	106	104	113	174	126	124	182	128	146	140	128	121	164	184	125	0	194	164	102	185	184	173	170	172	160	166	197	245	169	241
**CFSAN103796**	48	43	50	60	60	127	55	153	161	143	191	156	193	178	164	185	152	188	186	24	190	208	200	156	183	226	162	153	194	0	46	174	43	26	55	28	30	44	50	177	287	27	281
**CVM44454**	18	13	20	30	30	97	25	123	131	113	161	126	163	148	134	155	122	158	156	34	160	178	170	126	153	196	132	123	164	46	0	144	37	36	25	22	24	14	20	147	257	21	251
**CVM N1852085**	144	141	148	158	158	131	147	157	163	145	83	158	111	74	84	81	154	106	104	162	108	126	120	108	101	144	164	105	102	174	144	0	165	164	153	150	152	140	146	177	225	149	221
**CVM N1852198**	39	34	41	51	51	118	46	144	152	134	182	147	184	169	155	176	143	179	177	31	181	199	191	147	174	217	153	144	185	43	37	165	0	33	46	19	21	35	41	168	278	18	272
**CVM N1950949**	38	33	40	50	50	117	45	143	151	133	181	146	183	168	154	175	142	178	176	10	180	198	190	146	173	216	152	143	184	26	36	164	33	0	45	18	20	34	40	167	277	17	271
**FARPER-219**	27	22	29	39	39	106	34	132	140	122	170	135	172	157	143	164	131	167	165	43	169	187	179	135	162	205	141	132	173	55	25	153	46	45	0	31	33	23	29	156	266	30	260
**FSIS1502169**	24	19	26	36	36	103	31	129	137	119	167	132	169	154	140	161	128	164	162	16	166	184	176	132	159	202	138	129	170	28	22	150	19	18	31	0	6	20	26	153	263	3	257
**FSIS1502916**	26	21	28	38	38	105	33	131	139	121	169	134	171	156	142	163	130	166	164	18	168	186	178	134	161	204	140	131	172	30	24	152	21	20	33	6	0	22	28	155	265	5	259
**HUMV 13-6278**	14	11	18	28	28	93	21	119	127	109	157	122	159	144	130	151	118	154	152	32	156	174	166	122	149	192	128	119	160	44	14	140	35	34	23	20	22	0	12	143	253	19	247
**HUMV 15-5476**	20	17	24	34	34	99	27	125	131	115	163	128	165	150	136	157	124	160	158	38	160	180	170	128	155	194	134	125	166	50	20	146	41	40	29	26	28	12	0	149	257	25	253
**MRS-16/01939**	147	144	151	161	161	134	150	160	166	148	194	161	196	181	169	188	157	191	189	165	191	211	203	159	186	227	167	156	197	177	147	177	168	167	156	153	155	143	149	0	290	152	284
**MRS-17/00712**	257	254	261	271	271	246	260	272	276	260	242	273	244	229	217	236	269	239	237	275	237	259	251	223	234	273	279	220	245	287	257	225	278	277	266	263	265	253	257	290	0	262	176
**N55391**	23	18	25	35	35	102	30	128	136	118	166	131	168	153	139	160	127	163	161	15	165	183	175	131	158	201	137	128	169	27	21	149	18	17	30	3	5	19	25	152	262	0	256
**TR01**	251	248	255	265	265	238	254	264	270	252	238	265	240	225	213	232	261	235	233	269	237	255	249	219	230	273	271	216	241	281	251	221	272	271	260	257	259	247	253	284	176	256	0

Min: 0; max:290

Table S4. Oligonucleotides designed for this work and used for the assembly of the resistance regions (R1 and R2) of pESI-like plasmids found in *Salmonella enterica* serovar Infantis isolates from a Spanish hospital

Primer name	Sequence (5′-3′)
tauE fam	ACCTATAACGGAACCGATGACC
IS*1380* fam Flq	GGTGTCGGGAAATTTCGTTGAG
IS*903B*	AGTGGCTAATCAGCGAATGACC
tonB	CGATATTCAGGTCAACGATGCC
fosA3.3	CAGGGATTGAATCATCTGACGC
fosA3.2	CAGTAAGCTGAACTAACCCGTC
fosA3.1	GACGGGTTAGTTCAGCTTACTG
IS*91* fam.1	TGAAGTACTGCCTGAACAACCC
IS*91* fam.2	GCTTCAGGTGATCAAGGATCTG
floR.1	ATAGAGGCTCAACGTGAGTTGG
floR-2	CCAACTCACGTTGAGCCTCTAT
IS*6* fam	GCCCTTGAAATCATCCTTCGAC
aac(3)-IVa	TCCTGAAGAATGGTGCAGTGTC
DUF 1819 fam	AGGCAGCTTCATCAAGCTCTTG
drfA14.1	CAGAAGCCACTGATTGTAGGTC
drfA14.2	GACCTACAATCAGTGGCTTCTG
IS*3* fam	CACACCAAACACGTGCTGTATC
aph(3´)-Ia	ACCATGAGTGACGACTGAATCC
MFS-trans-flq	GTGTACGTCGGGTAATCATCTG
